# Targeted depletion of activated fibroblasts via [^177^Lu]Lu-FAPI radionuclide therapy ameliorates bleomycin-induced lung fibrosis

**DOI:** 10.3389/fimmu.2026.1884620

**Published:** 2026-07-29

**Authors:** Lin Sun, Zhaohua Li, Dan Fang, Shen Zheng, Hongkui Deng, Rong Mu

**Affiliations:** 1 Department of Rheumatology and Immunology, Center for Rare Disease, Peking University Third Hospital, Beijing, China; 2 Department of Rheumatology of the First Affiliated Hospital, Zhejiang University School of Medicine, Hangzhou, Zhejiang, China; 3 Tianjin Medical University Cancer Institute & Hospital, National Clinical Research Center for Cancer, Tianjin, China; 4 Department of Cell Biology, School of Basic Medical Sciences, Peking University Stem Cell Research Center, Peking University Health Science Center, Peking University, Beijing, China; 5 Changping Laboratory, Beijing, China

**Keywords:** fibroblast activation protein, Lutetium-177, macrophage polarization, pulmonary fibrosis, radiopharmaceuticals

## Abstract

**Introduction:**

Fibroblast activation protein (FAP)-targeted radiopharmaceuticals have been widely applied in oncologic imaging and therapy, while their application in pulmonary fibrosis remains underexplored. This study investigated the biodistribution and therapeutic potential of [^177^Lu]Lu-labeled FAP inhibitor (FAPI) in pulmonary fibrosis using two administered activity regimens.

**Methods:**

The therapeutic potential of an albumin-binder-conjugated FAPI radiopharmaceutical, [^177^Lu]Lu-TEFAPI-06 was evaluated in a murine model of bleomycin-induced pulmonary fibrosis. Targeting efficiency and pharmacokinetics were evaluated by small-animal PET/CT, SPECT/CT, and *ex vivo* biodistribution analyses. Therapeutic efficacy and tissue responses were then compared between two administered activity regimens.

**Results:**

[^177^Lu]Lu-TEFAPI-06 preferentially accumulated and was retained in fibrotic lungs with high uptake in FAP-positive lesions. The 11.1 MBq regimen significantly attenuated pulmonary fibrosis, reducing collagen deposition by approximately 50%, whereas the 18.5 MBq regimen failed to improve fibrosis and was accompanied by increased pulmonary macrophage infiltration and enrichment of Arg1-positive macrophages. Both regimens were well tolerated, with no detectable short-term toxicity.

**Discussion:**

This study provides proof-of-concept for FAP-targeted radionuclide therapy in experimental pulmonary fibrosis. Our findings suggest that distinct therapeutic outcomes are accompanied by different local tissue responses following treatment and support further evaluation of stromal-targeted radionuclide therapy for pulmonary fibrosis.

## 1 Introduction

Pathological fibrosis is a hallmark of numerous life-threatening diseases and contributes significantly to mortality (1). The activation and persistence of myofibroblasts are central drivers of the fibrotic cascade (2). During physiological wound healing, activated myofibroblasts are transient and undergo apoptosis upon repair completion (3). In contrast, in fibrotic conditions, these cells evade apoptosis, leading to excessive extracellular matrix (ECM) deposition and progressive tissue dysfunction (4, 5). Therefore, selective targeting and elimination of activated myofibroblasts has emerged as a promising therapeutic strategy.

FAP is highly expressed in activated rather than quiescent fibroblasts (6–11) and represents a marker of a major subset of pathogenic myofibroblasts in fibrotic diseases, with minimal expression in normal adult tissues (12–14). FAPI–based radiopharmaceuticals have recently emerged as a powerful platform for targeted imaging and therapy, particularly in the field of oncology (15, 16). These compounds offer high specificity, rapid internalization, and compatibility with therapeutic radionuclides such as Lutetium-177([^177^Lu]Lu) (17–21). The β-particle–mediated crossfire effect of [^177^Lu]Lu enables niche-targeted irradiation, potentially extending therapeutic impact beyond affecting individual FAP-expressing cells (22, 23). The established clinical safety profile of [^177^Lu]Lu-based therapies in humans provides a translational rationale for its investigation in fibrosis (24, 25).

Previously, an albumin-binder-conjugated FAPI radiopharmaceutical named [^177^Lu]Lu-TEFAPI-06 (19) was developed for cancer therapy, featuring optimized target retention. Here, we assess its therapeutic potential in a bleomycin-induced pulmonary fibrosis model. We provide proof-of-concept that among the two treatment regimens evaluated, the lower administered activity of [^177^Lu]Lu-TEFAPI-06 attenuated established fibrosis with a favorable short-term safety profile, whereas the higher administered activity did not improve fibrosis and was associated with increased macrophage infiltration. These findings thereby support the application of FAPI radionuclide therapy in pulmonary fibrosis and highlight activity-associated immune remodeling as an important consideration for broader application across diverse fibrotic diseases.

## 2 Materials and methods

### 2.1 Study design

This study was designed to eliminate activated fibroblasts by using FAPI radionuclide to treat pulmonary fibrosis. To determine the feasibility of the selective delivery of radionuclide therapy to fibrosis niche in pulmonary fibrosis by FAPI, we firstly verified FAP expression in lung tissues from interstitial lung disease (ILD) patients. *In vivo* specificity of [^68^Ga]Ga-FAPI-04 against FAP was verified by observing its uptake in bleomycin-induced pulmonary fibrosis model. To validate the therapeutic effect of FAP targeting radionuclide, we used a new albumin binder-conjugated FAPI radiopharmaceutical, [^177^Lu]Lu-TEFAPI-06, following a single dose of 11.1 MBq or 18.5 MBq on day 14 in bleomycin-induced lung fibrosis mice. The myofibroblast eliminating effect of [^177^Lu]Lu-TEFAPI-06 therapy was determined by IHC, immunofluorescence (IF) and TUNEL staining. Micro-CT and histological analyses were used to detect the pathological changes in pulmonary fibrosis after [^177^Lu]Lu-TEFAPI-06 radionuclide therapy.

### 2.2 Human lung tissues

Human FFPE tissues of pulmonary fibrosis (PF) were obtained from the Peking University Third Hospital (n=5). Fresh lung tissues were collected from non-fibrotic regions of lungs resected for lung cancer (adjacent normal tissue, n=6). The fresh tissues were formalin-fixed, embedded in paraffin, and sectioned at 4 µm thickness. The classification of PF was made by each subject’s primary pulmonologist according to American Thoracic Society/European Respiratory Society guidelines. The study on clinical samples was approved by the Ethics Committee of the Peking University Third Hospital ((2022) 142-01). All participants were provided with written informed consent before participating.

### 2.3 scRNA-seq analysis

Single-cell RNA sequencing data from pulmonary fibrosis and healthy lungs were obtained from the Gene Expression Omnibus (GEO) under accession number GSE136831 for bioinformatic reanalysis. Following canonical correlation analysis for dataset integration, cells were clustered and visualized using uniform manifold approximation and projection (UMAP) and t-distributed stochastic neighbor embedding (t-SNE). Cell clusters were subsequently annotated based on canonical marker genes with reference to the CellCards database.

### 2.4 Mouse model of bleomycin-induced lung fibrosis and [^177^Lu]Lu-TEFAPI-06 therapy

C57BL/6J mice were purchased from the Vital River Laboratory (China) and maintained on normal rodent chow. Mice were housed in a sterile environment on a standard 12-h light/dark cycle for the duration of the study. All animal procedures were approved by Animal Care and Use Committee of Peking University and performed in accordance with the Peking University Biomedical Ethics guidelines.

Pulmonary fibrosis was induced in mice via intratracheal instillation of bleomycin (BLM) at a dose of 1.5 U/kg. Age-matched control mice received an equal volume of sterile saline and served as controls. Animals were randomly assigned to four experimental groups, vehicle control (n=4), BLM-vehicle (n=6), BLM-11.1 MBq [^177^Lu]Lu-TEFAPI-06 (n=6), and BLM-18.5 MBq [^177^Lu]Lu-TEFAPI-06 (n=6). The day of BLM administration was designated as day 0. On day 14 post-instillation, different dose of [^177^Lu]Lu-TEFAPI-06 was administered by tail vein injection at 11.1 MBq or 18.5 MBq diluted in sterile saline to a final volume of 200 µL. All mice were humanely euthanized on day 21 for endpoint analysis.

### 2.5 Radionuclides

Both [^68^Ga]Ga-FAPI-04 imaging reagent and [^177^Lu]Lu-TEFAPI-06 radiopharmaceutical, which were described in previous publications (19, 20), were kindly provided by the laboratory of Zhibo Liu.

### 2.6 [^68^Ga]Ga-FAPI-04 PET/CT imaging

[^68^Ga]Ga-FAPI-04 PET imaging was performed using a nanoScan PET 122S small-animal Positron Emission Computed Tomography/Computed Tomography (PET/CT) imaging system (Mediso, The Hungary) on day 14 after BLM injection. Mice were anesthetized by inhalation of 2% isoflurane (Abbott Laboratories, Chicago, USA), and PET scanning was performed following tail-vein injection of 3.7 MBq [^68^Ga]Ga- FAPI-04 in 200 µL saline. Images were acquired 30 min after injection.

### 2.7 [^177^Lu]Lu-TEFAPI-06 SPECT/CT imaging and biodistribution

Single Photon Emission Computed Tomography/Computed Tomography (SPECT/CT) imaging was conducted using a Nano SPECT/CT scanner (Mediso, The Hungary). Each BLM-treated mouse was injected with 18.5 MBq of [^177^Lu]Lu-TEFAPI-06 through the tail vein under anesthesia (2% isoflurane in oxygen). The mice were imaged at 24 h, 48 h, and 120 h after injection. For biodistribution studies, the mice were injected with [^177^Lu]Lu-TEFAPI-06 (18.5 MBq) as described above. At 24 h and 72 h, the mice were euthanized by CO_2_ inhalation. Blood was obtained immediately from the inner canthus. The organs/tissues of interest were collected, weighed and counted using an automated gamma counter.

### 2.8 Micro-computed tomography

Mouse lungs were scanned longitudinally using Quantum FX Micro-CT (PerkinElmer, Inc. Waltham, MA, USA) on days 14 and day 21. Each mouse was anesthetized by inhalation of 2% isoflurane and positioned inside the micro-CT imager. Images were acquired using an X-ray tube voltage of 90 kV and current of 160 µA over a total angle of 360°, with a 4.5-min scan time in high speed scan mode with respiratory gating. The measurement is expressed in Hounsfield Units (HU).

### 2.9 Histological analysis

Immunohistochemistry (IHC) staining of human and mouse lung samples was performed by using the following antibodies: anti-human FAP (1:500, Abcam, ab53066), anti-human α-SMA (1:300, Cell Signaling Technology, 19245s), anti-mouse FAP (1:100, Abcam, ab218164), anti-mouse α-SMA (1:200, Cell Signaling Technology, 19245s), anti-mouse F4/80 (1:500, Abcam, ab300421), anti-mouse CD3 (1:50, Abcam, ab828), anti-mouse iNOS (1:500, Servicebio, GB11119-50) and anti-mouse Arg1 (1:500, Servicebio, GB115724-50). Masson’s trichrome staining was used for the detection of collagen fibers in lung tissues. Fixation and paraffin-embedding of sections were performed following the product manual of the Masson’s trichrome staining kit (Solarbio science & technology, catalog no. G1346). Collagen in lung sections was also visualized with Picro-Sirius Red staining kit (Beijing Leagene Biotechnology, catalog no. DC0041) following a standard protocol. The extent of fibrosis was then quantified by measuring the percentage of Sirius Red-positive area. To evaluate the safety of radionuclide therapy with [^177^Lu]Lu-TEFAPI-06, the main organs including the lung, liver, heart, spleen, kidney of mice treated with saline or [^177^Lu]Lu-TEFAPI-06 were subjected to hematoxylin and eosin staining. QuPath-0.6.0 was used for imaging analysis.

### 2.10 TUNEL assay

The apoptotic myofibroblasts in the fibrotic lung of mice were detected using a terminal deoxynucleotidyl transferase–mediated dUTP nick-end labeling (TUNEL) apoptosis detection kit (Applygen, catalog no. C0001). The TUNEL assay was carried out on 5 μm-thick lung sections. More than 10 fields per section in each group were investigated using an Olympus BX-61 microscope, and the images were captured using a DP-70 digital camera (Olympus, Japan). In each section, three fields were selected for examination, and the number of apoptotic myofibroblasts was counted in each high-power field (200×).

### 2.11 Analysis of peripheral blood

Peripheral blood was drawn from the ocular artery of each mouse using a 5.5-mm animal lancet and placed in anticoagulant-coated tubes (Solarbio). The blood count was measured using an XN-1000V hematology analyzer (Sysmex Corp, Kobe, Japan).

### 2.12 Statistical analysis

Data are expressed as the mean ± standard error of the mean (SEM) and were statistically analyzed using the GraphPad Prism 9.0 software. For comparisons of two groups, P values were calculated using a two-tailed unpaired Student’s t-test for Gaussian distributed data when the variances were similar. For comparisons of three groups or more, P values were calculated using one-way ANOVA for normally distributed data followed by Tukey’s multiple comparisons test. For comparisons of repeated measurements across different time points among three or more groups, P values were calculated using two-way ANOVA with Šídák’s multiple comparisons test. Survival analysis used log-rank test. Statistical significance was defined as P<0.05.

## 3 Results

### 3.1 FAP is enriched in pathogenic fibroblasts

**Figure 1:**
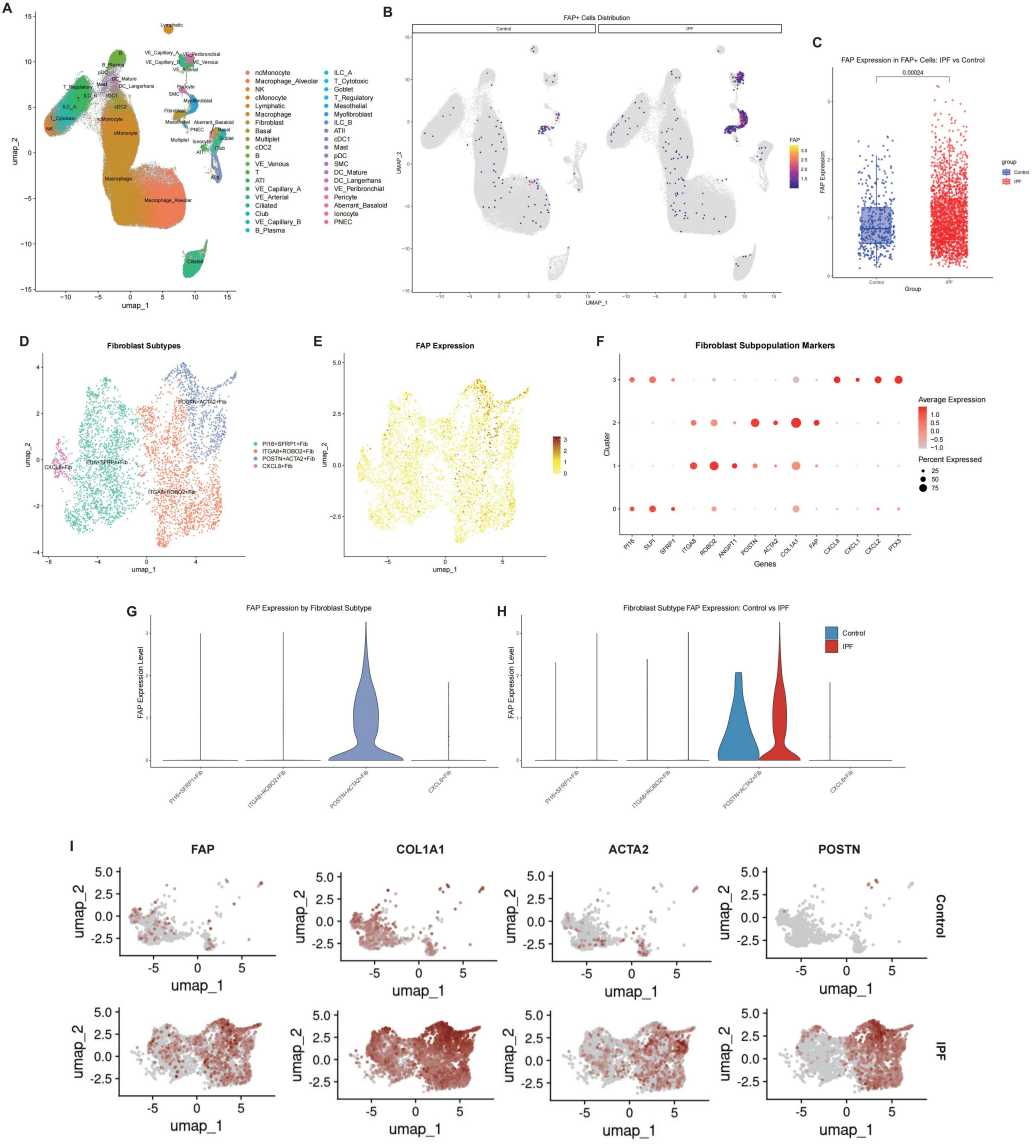
FAP identifies a pathogenic fibroblast subset in idiopathic pulmonary fibrosis. **(A)** UMAP of scRNA-seq data from healthy donors (HC, n=26) and patients with idiopathic pulmonary fibrosis (IPF, n=30). **(B)** Feature plot showing FAP expression in HC and IPF lung tissues. **(C)** Quantification of FAP-positive cells in HC and IPF samples. **(D)** UMAP visualization of fibroblast subclusters identified from the integrated scRNA-seq dataset. **(E)** Feature plot showing FAP expression across fibroblast subsets. **(F)** Fibroblast subpopulation markers, Cluster 0: PI16^+^SFRP1^+^Fib; Cluster 1: ITGAB^+^ROBO2^+^Fib; Cluster 2: POSTN^+^ACAT2^+^Fib; and Cluster 3: CXCL8^+^Fib. **(G)** Relative FAP expression across the four fibroblast subsets. **(H)** Comparison of FAP expression in fibroblast subsets between HC and IPF samples. **(I)** Differential expression of selected profibrotic genes in HC and IPF samples.

**Figure 2:**
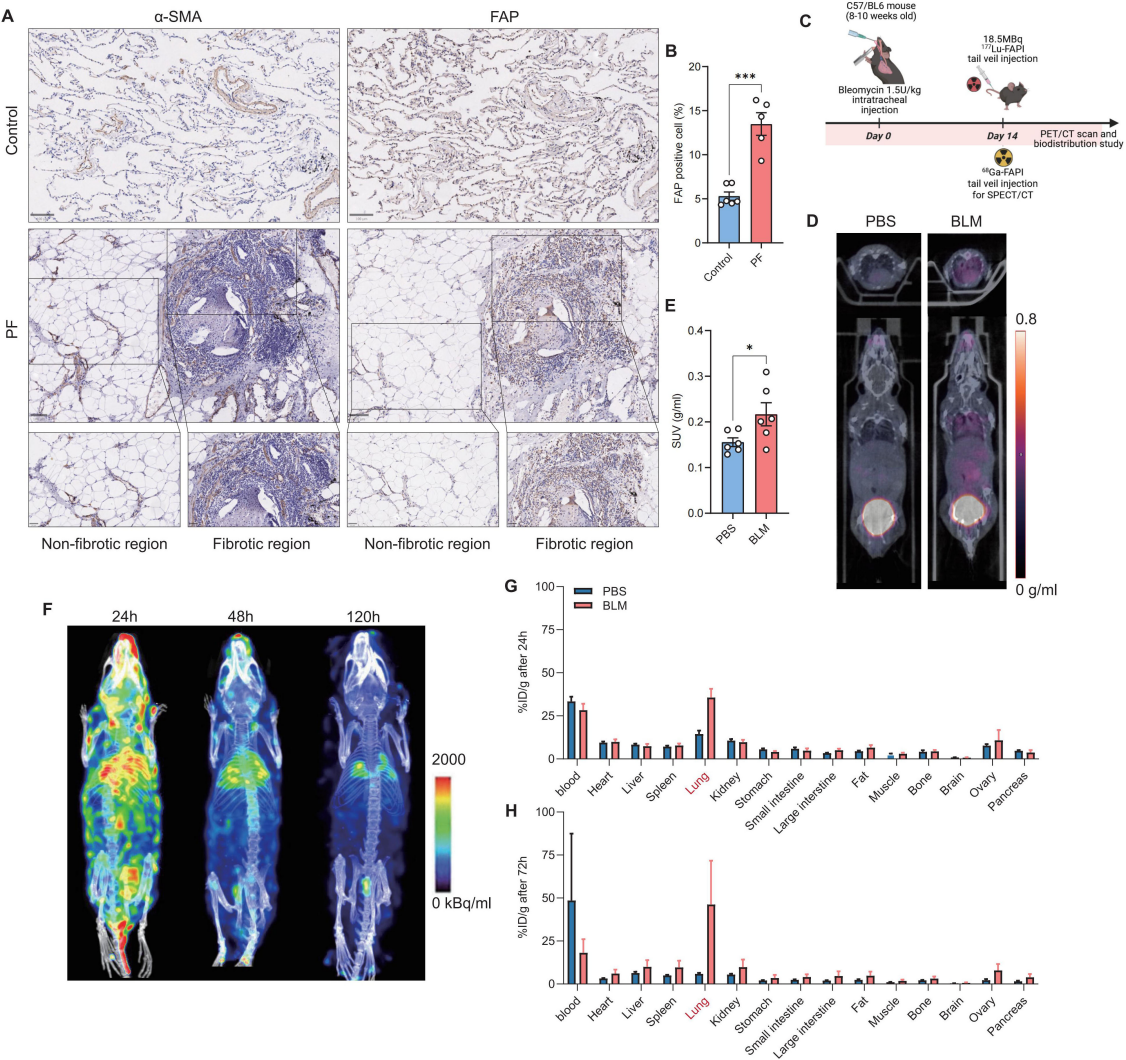
FAP is enriched in fibrotic niches and enables FAPI-based molecular imaging of pulmonary fibrosis. **(A)** Representative images of FAP expression in fibrotic and non-fibrotic lung regions from patients with pulmonary fibrosis and healthy donors. Scale bar, 100μm. **(B)** Quantification of FAP-positive cells in lung tissues from healthy donors (n=6) and pulmonary fibrosis patients (n=5). Data are presented as mean ± SEM and were analyzed using an unpaired t-test. ***P < 0.001. **(C)** Experimental schematic of the BLM-induced pulmonary fibrosis mouse model, followed by [^68^Ga]Ga-FAPI-04 PET/CT imaging, [^177^Lu]Lu-TEFAPI-06 SPECT/CT imaging, and biodistribution analysis. **(D)** Transverse (top) and coronal (bottom) PET/CT images showing [^68^Ga]Ga-FAPI-04 uptake in the lungs of saline-treated mice (n=6) and BLM-treated mice (n=6) at day 14 after BLM administration. **(E)** Quantification of SUV values of [^68^Ga]Ga-FAPI uptake in mouse lungs. Data are presented as mean ± SEM and Data were analyzed using an unpaired t-test. *P < 0.05. **(F)** Representative SPECT/CT images of [^177^Lu]Lu-TEFAPI-06 in saline and BLM-treated mice at 24 h, 48 h and 120 h post injection. **(G–H)** Biodistribution of [^177^Lu]Lu-TEFAPI-06 at 24 h (PBS, n=5, BLM, n=4) and 72 h (PBS, n=5, BLM, n=4) post injection in selected organs.

To define the cellular distribution of FAP in pulmonary fibrosis, we reanalyzed publicly available single-cell RNA sequencing data from fibrotic and healthy human lungs (GSE136831). The proportion of FAP^+^ cells was markedly increased in fibrotic lungs compared with healthy controls (**Figures 1A–C**). Cell-type annotation revealed that FAP expression was largely restricted to the fibroblast lineage. Further fibroblast subcluster analysis demonstrated that FAP was highly enriched in a subset of fibroblasts co-expressing POSTN and ACTA2 (**Figures 1D–H**). This population displayed strong expression of profibrotic and extracellular matrix associated genes, including ACTA2, COL1A1, POSTN, and FAP (**Figure 1I**), consistent with a highly activated matrix-producing myofibroblast phenotype. Immunohistochemistry (IHC) further confirmed significantly elevated FAP expression in lung tissues from patients with ILD compared to normal controls (**Figures 2A, B**). FAP-positive areas were co-localized with α-smooth muscle actin (α-SMA)-positive fibrotic niches (**Figure 2A**), supporting its potential for targeted delivery of FAPI-based radionuclides.

### 3.2 *In vivo* targeting of pulmonary fibrosis by [^68^Ga]Ga-FAPI-04 and [^177^Lu]Lu-TEFAPI-06

To evaluate *in vivo* targeting of fibrotic lesions, we performed PET/CT imaging using [^68^Ga]Ga-FAPI-04 in a bleomycin (BLM)-induced murine pulmonary fibrosis model. Fourteen days after BLM administration, PET/CT imaging revealed robust and specific tracer accumulation in fibrotic lungs (**Figures 2C–E**). Quantitative analysis showed significantly higher tracer uptake in BLM-treated mice (SUVmean = 0.22 ± 0.03) compared with controls (SUVmean = 0.16 ± 0.01, *P*< 0.05).

To further assess the potential for radioligand therapy, longitudinal SPECT/CT imaging was performed using [^177^Lu]Lu-FAPI-06 in the pulmonary fibrosis model at 24 h, 48 h, and 120 h post-injection after BLM injury. The tracer exhibited prolonged pulmonary retention in fibrotic lungs up to 120 h, while clearing rapidly from healthy organs (**Figure 2F**). The lung uptake in the BLM group increased from 35.615 ± 5.082%ID/g (24 h) to 46.207 ± 25.476%ID/g (72 h), contrasting with the rapid clearance seen in controls (14.467 ± 2.021%ID/g to 5.849± 0.637%ID/g) (**Figures 2G, H**). These results support the feasibility of lung-targeted radionuclide therapy in fibrotic disease.

### 3.3 [^177^Lu]Lu-TEFAPI-06 radionuclide therapy promotes myofibroblast apoptosis *in vivo*

**Figure 3:**
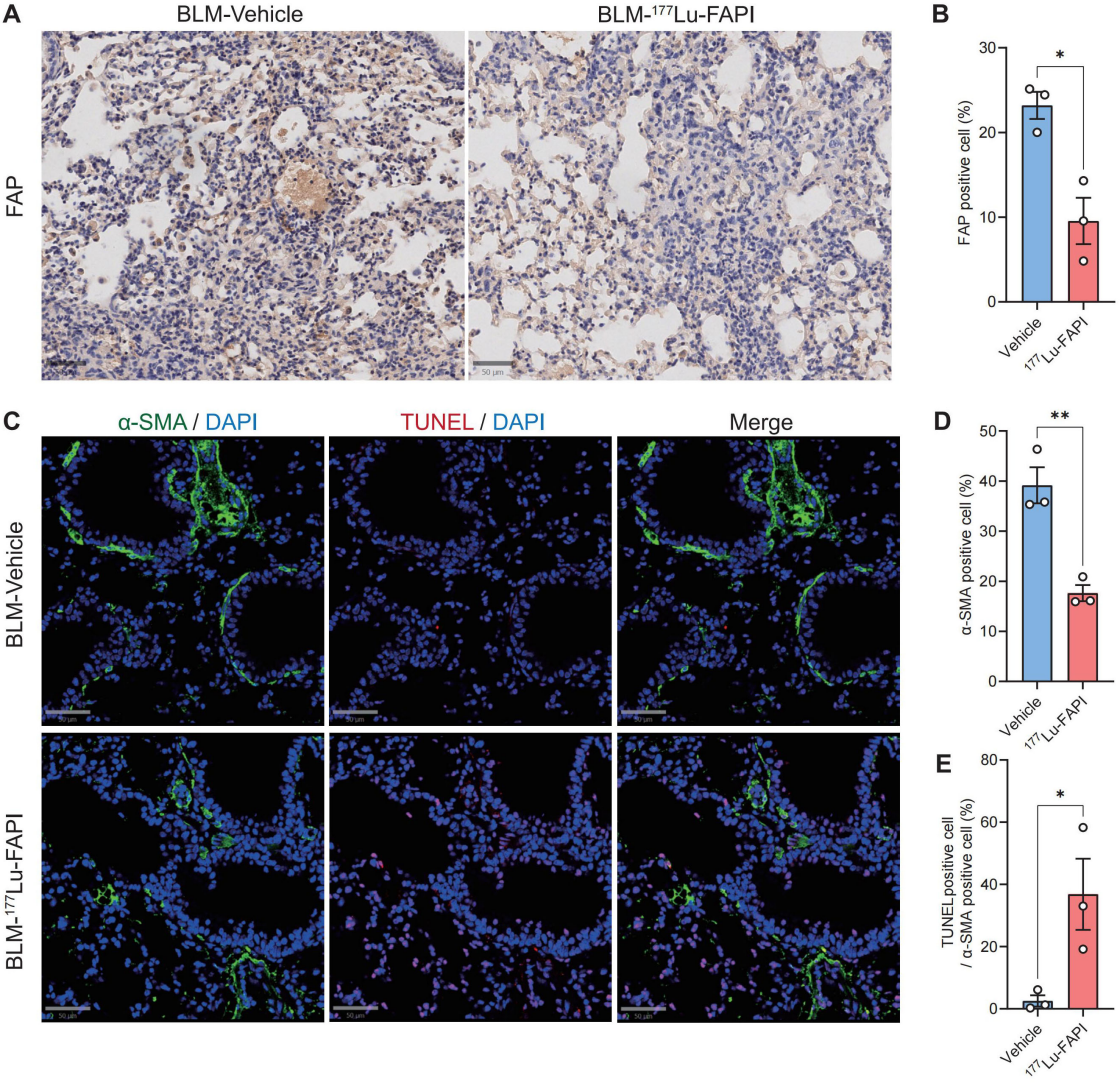
[^177^Lu]Lu-TEFAPI-06 radionuclide therapy promotes myofibroblast apoptosis *in vivo*. **(A)**. Representative images of FAP expression on day 3 post injection of saline or [^177^Lu]Lu-TEFAPI-06 in the lungs of each treatment group. Scale bar, 50 μm. **(B)** Quantification of FAP-positive cells in each treatment group shown as mean ± SEM and were analyzed using an unpaired t-test. *P < 0.05. **(C)** Representative α-SMA and TUNEL-stained fixed tissue section (Scale bar, 50 μm). α-SMA was shown in green, TUNEL-positive staining was depicted in red, nuclei were observed by staining DAPI. **(D)** Quantitation of the proportion of α-SMA+ cells in different groups shown as mean ± SEM (n=3 per each group). Data were analyzed using an unpaired t-test (**P < 0.01). **(E)** Quantitation of the proportion of TUNEL+/α-SMA+ double-positive cells in the different groups shown as mean ± SEM (n=3 per each group). Data were analyzed using an unpaired t-test (*P < 0.05).

Having confirmed the specific uptake of [^177^Lu]Lu-TEFAPI-06 in fibrotic lungs, we next assessed its ability to deplete myofibroblasts *in vivo*. A single administration of [¹⁷⁷Lu]Lu-TEFAPI-06 significantly reduced the proportion of FAP-positive cells by 58.8% within 3 days, as assessed by IHC, compared with vehicle-treated controls (**Figures 3A, B**). Immunofluorescence (IF) and TUNEL co-staining revealed an approximately 55.0% reduction in α-SMA–positive myofibroblasts, accompanied by a marked 14.5-fold increase in apoptotic myofibroblasts, as indicated by the number of TUNEL^+^/α-SMA^+^ double-positive cells (**Figures 3C–E**). These data demonstrate that [^177^Lu]Lu-TEFAPI-06 radionuclide therapy effectively eliminates activated myofibroblasts through induction of apoptosis.

### 3.4 The 11.1 MBq [^177^Lu]Lu-TEFAPI-06 therapy attenuates pulmonary fibrosis

**Figure 4:**
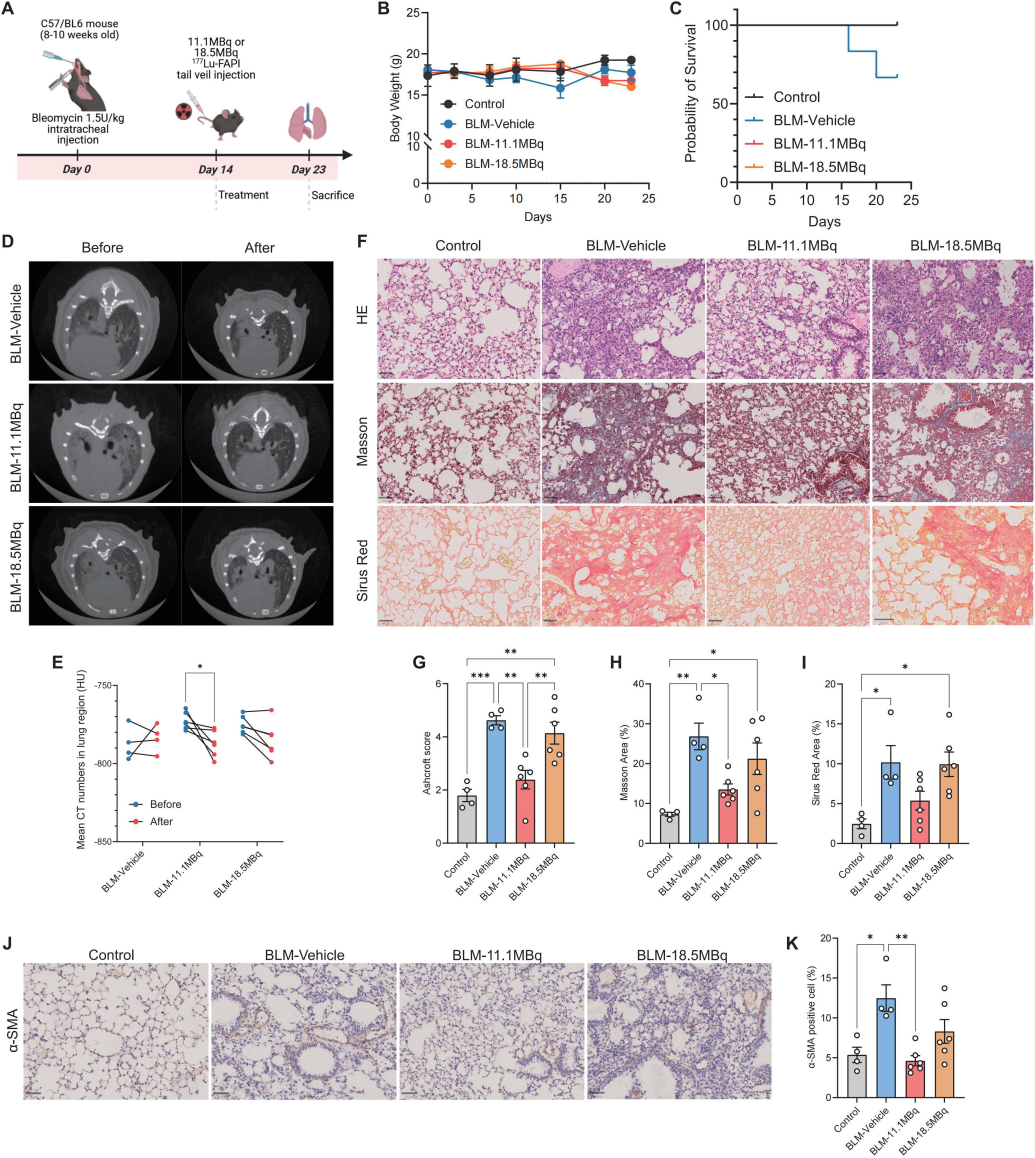
Lower-activity [^177^Lu]Lu-TEFAPI-06 ameliorates pulmonary fibrosis *in vivo*. **(A)** Schematic representation of the experimental protocol for the induction and therapeutic intervention of bleomycin-induced lung fibrosis mouse model. **(B)** Body weight changes observed after bleomycin administration. **(C)** Kaplan-Meier survival analysis. P values were determined by log-rank test. **(D)** Representative micro-CT images from saline mice and BLM-induced mice treated with vehicle or [^177^Lu]Lu-TEFAPI-06 (11.1 or 18.5 MBq) at days 14 (before treatment) and 21 (after treatment) following bleomycin administration. **(E)** Paired changes of CT attenuation voxel of BLM-induced mice treated with vehicle (n=4), 11.1 MBq (n=6) and 18.5 MBq (n=6) on day 14 and day 21 were analyzed. **(F)** Representative histological images of the lung sections stained by hematoxylin and eosin (H&E), Masson, and Sirus Red in each experimental group. Scale bar, 50 μm. G-I. The fibrotic severity was evaluated using the Ashcroft score **(G)**, percentage of masson area **(H)** and percentage of sirus red area **(I)** in each experimental group (Control group, n=4; BLM-vehicle treated group, n=4; and [^177^Lu]Lu-TEFAPI-06 11.1 MBq and 18.5 MBq treated group, n=6). **(J)** Myofibroblasts labeled with α-SMA in the lungs of each treatment group were shown. Scale bar, 50μm. **(K)** The percentage of α-SMA+ myofibroblasts in the lungs was calculated. Data were analyzed using one-way ANOVA, followed by Tukey’s *post hoc* test (*P < 0.05, **P < 0.01, ***P < 0.001).

Mice with BLM-induced established fibrosis received a single intravenous injection of [^177^Lu]Lu-TEFAPI-06 at 11.1 MBq or 18.5 MBq, or saline as a vehicle control (**Figure 4A**). Body weight changes were comparable across all groups (**Figure 4B**). All treated mice survived to endpoint, whereas the vehicle group exhibited a mortality rate of 33.3% (**Figure 4C**). The 11.1 MBq group showed a significant decrease in Micro-CT density at day 21 compared with day 14 (-787.30 ± 3.47 HU vs. -772.70 ± 2.26 HU; *P* < 0.05), indicating reduced consolidation (**Figures 4D, E**) and a 48.3% reduction in the Ashcroft histology score versus controls (**Figures 4F, G**). Consistent with these findings, collagen deposition was reduced by 49.6%, as assessed by Masson’s trichrome and Sirius Red staining (**Figures 4F, H, I**) and α-SMA–positive myofibroblasts were decreased by 62.9% in the 11.1 MBq group (**Figures 4J, K**). In contrast, the 18.5 MBq group showed no significant improvement in these parameters.

### 3.5 The 18.5 MBq [^177^Lu]Lu-TEFAPI-06 therapy is associated with an Arg1-positive macrophage-enriched microenvironment

**Figure 5:**
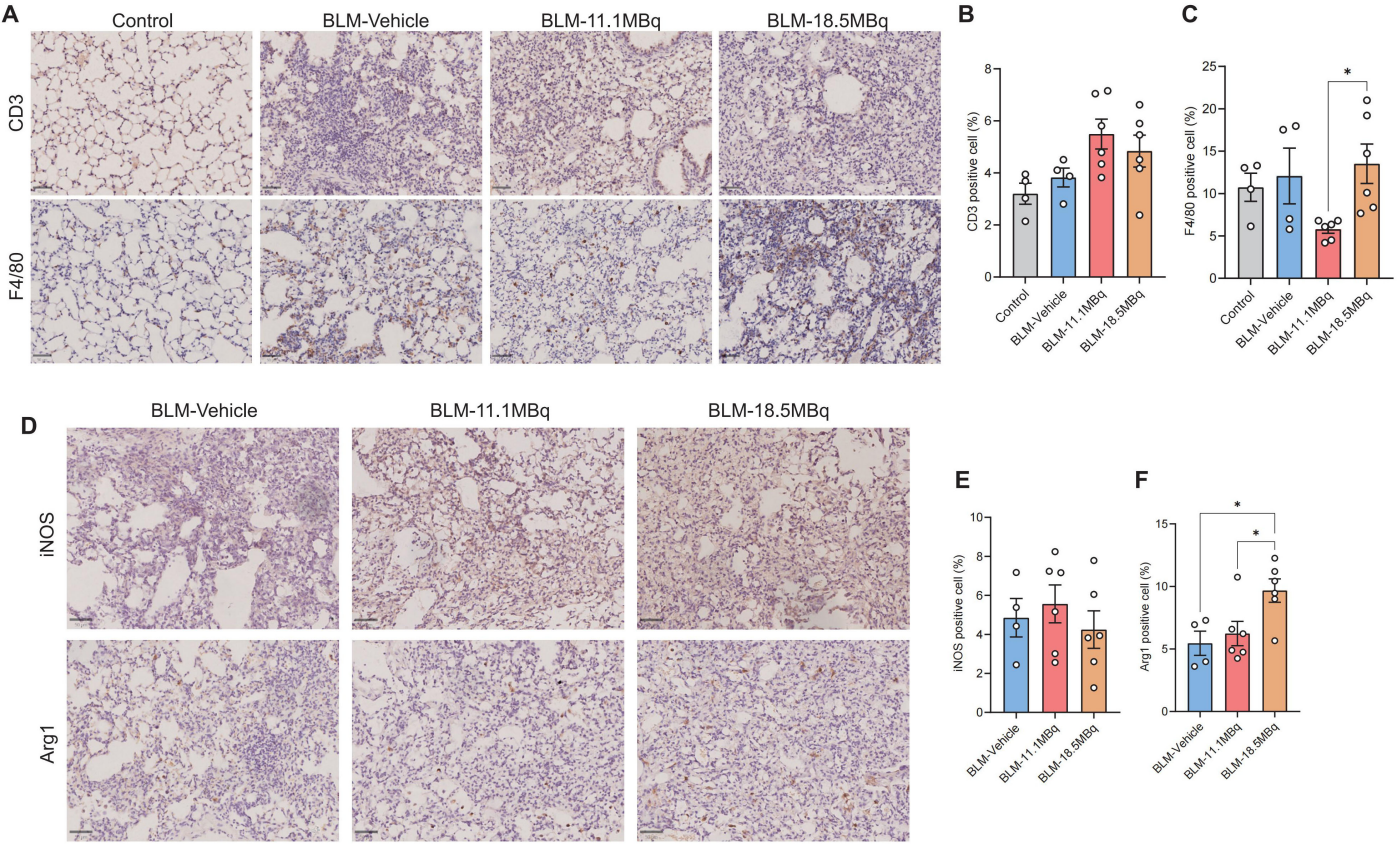
Higher-activity [^177^Lu]Lu-TEFAPI-06 is associated with an Arg1-positive macrophage-enriched microenvironment. **(A)** Representative CD3 and F4/80 staining in the lungs of each treatment group were shown. Scale bar, 50μm. B-C. Quantification of CD3-positive **(B)** and F4/80-positive **(C)** areas (Control, n=4; BLM-vehicle, n=4; and [^177^Lu]Lu-TEFAPI-06-treated groups, n=6 per group). **(D)** Representative iNOS and Arg1 staining. Scale bar, 50 μm. E-F. Quantification of iNOS-positive **(E)** and Arg1-positive **(F)** areas (BLM-vehicle, n=4; and [^177^Lu]Lu-TEFAPI-06-treated groups, n=6 per group). Data were analyzed using one-way ANOVA, followed by Tukey’s multiple-comparisons test, (*P < 0.05).

Investigation of the immune microenvironment revealed that, compared with the 11.1 MBq regimen, the 18.5 MBq regimen was associated with increased pulmonary infiltration of F4/80+ macrophages (13.51 ± 2.33% vs 5.79 ± 0.47%; *P* < 0.05), without affecting CD3^+^ T cells (**Figures 5A–C**). These results suggest that the 18.5 MBq regimen was accompanied by enhanced macrophage recruitment. Phenotypic analysis further identified a specific enrichment of Arg1-positive, M2-like macrophages in the 18.5 MBq group compared with the 11.1 MBq group (9.67 ± 0.93% vs 6.24 ± 0.97%; *P* < 0.05), with no change in iNOS-positive macrophages (**Figures 5D–F**). These findings indicate an association between the higher administered activity and an Arg1-positive macrophage-enriched microenvironment, without establishing a causal relationship.

### 3.6 [^177^Lu]Lu-TEFAPI-06 therapy shows no detectable short-term systemic toxicity

**Figure 6:**
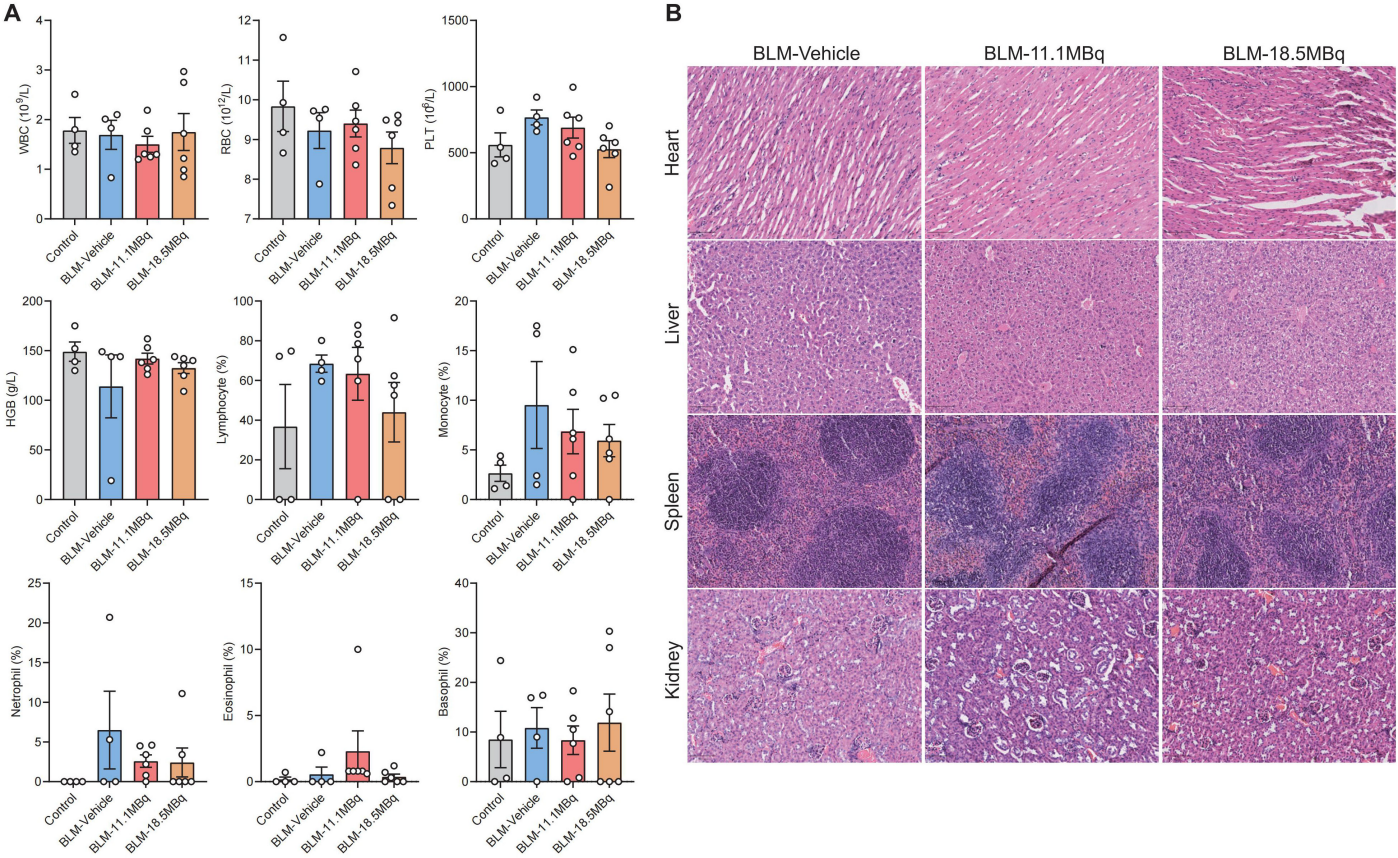
**(A)** Counts or percentages of the peripheral blood cells from BLM-vehicle treated group (n=4), and [¹⁷⁷Lu]Lu-TEFAPI-06 11.1 MBq and 18.5 MBq treated group (n=6). **(B)** H&E staining of tissue sections from mice described in **(A)**. Scale bar, 100mm.

Short-term toxicity assessment showed no significant abnormalities in peripheral blood cell counts (**Figure 6A**), and histopathology of major organs, including the heart, liver, kidney, and spleen revealed no appreciable treatment-related lesions following either the 11.1 or 18.5 MBq [^177^Lu]Lu-TEFAPI-06 treatment (**Figure 6B**). Within the short observation period, these findings indicate that [^177^Lu]Lu-TEFAPI-06 therapy showed no detectable short-term systemic toxicity under the conditions evaluated.

## 4 Discussion

In the present study, we provide proof-of-concept that FAP-targeted [^177^Lu]Lu-TEFAPI-06 radionuclide therapy can attenuate experimental pulmonary fibrosis. Using two administered activity regimens of the same radiopharmaceutical, we found that only the lower-activity regimen demonstrated anti-fibrotic efficacy, whereas the higher-activity regimen failed to improve fibrosis and was accompanied by increased pulmonary macrophage infiltration. These observations suggest that the therapeutic outcome of FAP-targeted radionuclide therapy may depend not only on effective targeting of activated fibroblasts but also on the accompanying local tissue response after treatment. Building upon the established role of FAPI molecular imaging in pulmonary fibrosis, our study extends FAPI from target visualization to therapeutic intervention and supports further development of stromal-targeted radionuclide therapy for fibroblast-driven diseases.

Among the local tissue changes observed after treatment, the macrophage response was particularly notable. The higher-activity regimen was associated with increased pulmonary infiltration of F4/80-positive macrophages and enrichment of Arg1-positive macrophages. Macrophages are central regulators of extracellular matrix remodeling, inflammatory resolution, and tissue repair. Persistent enrichment of alternatively activated macrophages has been implicated in progressive pulmonary fibrosis (26, 27). In parallel, previous studies of targeted radionuclide therapy have shown that different administered activities can generate distinct immune signatures within irradiated tissues, including alterations in myeloid-cell composition (28). These findings suggest that activity-associated changes in the local tissue response may contribute to the different therapeutic outcomes observed following FAP-targeted radionuclide therapy.

Clinical development of FAPI in fibrotic disease has thus far focused predominantly on molecular imaging. In contrast, therapeutic application of FAP-targeted radionuclide therapy in pulmonary fibrosis remains largely unexplored. Several studies have shown that pulmonary FAPI uptake reflects fibroblast activation and correlates with disease activity and progression in interstitial lung disease (17, 18, 29), consistent with our finding that [^68^Ga]Ga-FAPI-04 visualized FAP-positive fibrotic lung. Consistent with our imaging findings, Sun et al. used longitudinal [^68^Ga]Ga-FAPI-04 PET/CT in a bleomycin-induced C57BL/6 model and observed progressive pulmonary tracer uptake that peaked at 4 weeks and paralleled histopathological fibrosis (30). Whereas that study focused on imaging disease evolution and monitoring conventional antifibrotic treatment, our study administered [^177^Lu]Lu-TEFAPI-06 on day 14 and evaluated direct FAP-targeted radionuclide therapy, thereby extending FAPI from longitudinal assessment to therapeutic intervention. Current experience with [^177^Lu]Lu-FAPI has been derived primarily from oncology. Preclinical studies with [^177^Lu]Lu-TEFAPI-06 have demonstrated prolonged target retention and effective tumor growth inhibition (19). Similarly, dose-ranging experiments with [^177^Lu]Lu-FAPI-46 in pancreatic cancer xenografts revealed enhanced tumor suppression at higher administered activities (20). Early clinical case series summarized by Privé et al. (21) and a subsequent first-in-human dose-escalation study of [^177^Lu]Lu-FAPI-XT (24) support feasibility in advanced cancers. Collectively, these studies establish the translational feasibility of FAP-targeted radionuclide therapy but do not support a universal activity–efficacy relationship across different FAPI agents or disease settings. In pulmonary fibrosis, treatment outcome is likely influenced not only by target engagement but also by disease-specific local tissue responses. Accordingly, the distinct outcomes observed between the two administered activity regimens in the present study should be interpreted within the biological context of this model rather than as evidence of a general activity-response relationship.

This study has two important limitations. First, the bleomycin model represents an acute and partially resolving fibrotic response that does not fully recapitulate the chronic and progressive nature of human pulmonary fibrosis. Second, no organ- or lesion-level absorbed-dose calculation was performed for either regimen, and parallel biodistribution data were unavailable for the 11.1 MBq regimen. Accordingly, the differences observed between the two administered activity regimens should be interpreted as regimen-associated biological responses rather than as a formal absorbed-dose-response relationship. Future studies should incorporate parallel biodistribution for both administered activities, voxel-based lung dosimetry, lesion-level absorbed-dose estimation, and chronic fibrosis models to strengthen the translational potential of these findings.

In conclusion, our study provides proof-of-concept for FAP-targeted radionuclide therapy in experimental pulmonary fibrosis. These findings support further development of stromal-targeted theranostic strategies and highlight the importance of local tissue responses in shaping therapeutic outcome.

## Data Availability

The original contributions presented in the study are included in the article. The publicly available scRNA-seq dataset analyzed in this study is available in the Gene Expression Omnibus under accession number GSE136831. Further inquiries can be directed to the corresponding authors.
